# A proof of concept for microcirculation monitoring using machine learning based hyperspectral imaging in critically ill patients: a monocentric observational study

**DOI:** 10.1186/s13054-024-05023-w

**Published:** 2024-07-10

**Authors:** Judith Kohnke, Kevin Pattberg, Felix Nensa, Henning Kuhlmann, Thorsten Brenner, Karsten Schmidt, René Hosch, Florian Espeter

**Affiliations:** 1 Institute of Diagnostic and Interventional Radiology and Neuroradiology, University Hospital Essen, Essen, Germany; 2 Institute for Artificial Intelligence in Medicine (IKIM), University Hospital Essen, Essen, Germany; 3 Department of Anesthesiology and Intensive Care Medicine, University Hospital Essen, University Duisburg-Essen, Essen, Germany

## Abstract

**Background:**

Impaired microcirculation is a cornerstone of sepsis development and leads to reduced tissue oxygenation, influenced by fluid and catecholamine administration during treatment. Hyperspectral imaging (HSI) is a non-invasive bedside technology for visualizing physicochemical tissue characteristics. Machine learning (ML) for skin HSI might offer an automated approach for bedside microcirculation assessment, providing an individualized tissue fingerprint of critically ill patients in intensive care. The study aimed to determine if machine learning could be utilized to automatically identify regions of interest (ROIs) in the hand, thereby distinguishing between healthy individuals and critically ill patients with sepsis using HSI.

**Methods:**

HSI raw data from 75 critically ill sepsis patients and from 30 healthy controls were recorded using TIVITA® Tissue System and analyzed using an automated ML approach. Additionally, patients were divided into two groups based on their SOFA scores for further subanalysis: less severely ill (SOFA ≤ 5) and severely ill (SOFA > 5). The analysis of the HSI raw data was fully-automated using MediaPipe for ROI detection (palm and fingertips) and feature extraction. HSI Features were statistically analyzed to highlight relevant wavelength combinations using Mann–Whitney-U test and Benjamini, Krieger, and Yekutieli (BKY) correction. In addition, Random Forest models were trained using bootstrapping, and feature importances were determined to gain insights regarding the wavelength importance for a model decision.

**Results:**

An automated pipeline for generating ROIs and HSI feature extraction was successfully established. HSI raw data analysis accurately distinguished healthy controls from sepsis patients. Wavelengths at the fingertips differed in the ranges of 575–695 nm and 840–1000 nm. For the palm, significant differences were observed in the range of 925–1000 nm. Feature importance plots indicated relevant information in the same wavelength ranges. Combining palm and fingertip analysis provided the highest reliability, with an AUC of 0.92 to distinguish between sepsis patients and healthy controls.

**Conclusion:**

Based on this proof of concept, the integration of automated and standardized ROIs along with automated skin HSI analyzes, was able to differentiate between healthy individuals and patients with sepsis. This approach offers a reliable and objective assessment of skin microcirculation, facilitating the rapid identification of critically ill patients.

## Introduction

Sepsis and septic shock persist as formidable, life-threatening conditions where the survival of critically ill patients hinges upon effective hemodynamic therapy [1, 2]. Hemodynamic therapy's primary objective is to ensure tissue oxygen supply to sustain metabolic functions [3]. Despite extensive research, bedside microcirculation monitoring technology has not yet been routinely integrated into clinical practice in intensive care medicine [3–5]. The future challenge revolves around facilitating non-invasive, straightforward, and reliable bedside assessment, along with quantitative analysis of the microcirculation to enable personalized tissue perfusion guided therapy in critically ill patients [3]. To bridge the chasm between research and clinical application in microcirculation monitoring, machine learning (ML)-based analysis has emerged as a compelling avenue [1, 6]. Hyperspectral Imaging (HSI), a non-invasive optical imaging technology offers great potential for automated image processing and data analysis to efficiently distinguish between different tissue types or changes in tissue condition using artificial intelligence [7, 8]. Few studies have examined skin HSI in critical care or perioperative settings, but the results suggest that bedside skin HSI technology may expand the possibilities for microcirculation monitoring in the future [9–12]. Initial studies on skin HSI in intensive care medicine showed that HSI can clearly distinguish septic patients from healthy volunteers [9, 11]. HSI can provide spatially visualized information on the quality of oxygenation, adequacy of perfusion and water content of the tissue area under investigation [8, 13–15]. In addition to its ability to detect clinically relevant microcirculatory disorders, initial clinical and experimental studies have suggested that HSI monitoring may provide feedback on tissue perfusion and oxygenation during resuscitation therapy, including detection of adverse fluid and vasopressor effects [9–12, 16, 17].

Our group previously investigated the association between the severity of organ dysfunction, as assessed by the Sequential Organ Failure Assessment (SOFA) scoring, and palmar skin HSI in critically ill COVID-19 patients in a monocentric observational study. Based on the HSI parameters provided by the TIVITA® Tissue camera system, the most prominent observations were the persistent reduction in oxygenation parameters and evidence of increased tissue water content. Further regression analyses showed a relationship between HSI perfusion and oxygenation parameters with organ dysfunction severity as well as associations to vasopressor support, lactate levels and arterial oxygen saturation [12].

Studier-Fischer et al. have previously demonstrated the potential of automated organ recognition using organ-specific HSI patterns or spectral fingerprints in animal experiments using raw spectral data acquired with the TIVITA® device and machine learning analysis [18].

We hypothesized that the potential of the HSI technology used to monitor skin microcirculation in critical care has not yet been fully exploited. The available raw spectral data on which the algorithms for the TIVITA® parameters are based may contain clinically relevant but methodologically underutilized information, which could further improve HSI applicability as microcirculation monitoring. This study was conducted as a proof of concept to investigate whether automatic identification of regions of interest (ROIs) in the hand and machine learning analysis of raw spectral data could be used to distinguish between healthy controls and patients with sepsis.

## Material and methods

**Fig. 1:**
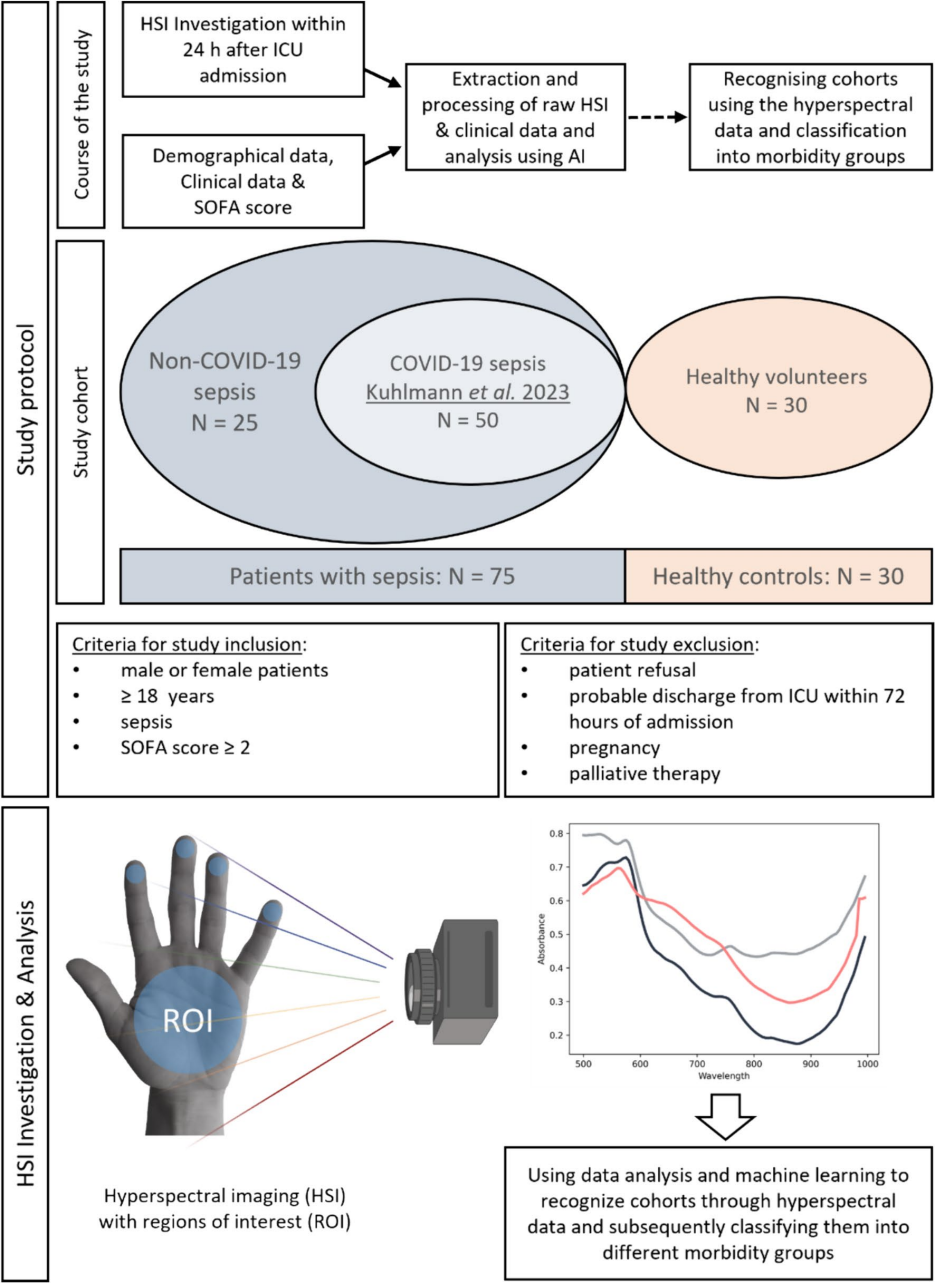
HSI measurements of the inside of the patient's hand are carried out within 24 h after admission to ICU. At the same time, demographic data, clinical data and the SOFA score are recorded in order to assess the patient's state of health. Automatic ROI detection and a machine learning-based analysis of the raw HSI data were performed

This monocentric, prospective observational study was designed during the COVID-19 pandemic to include critically ill patients with COVID-19 [12]. To include septic, critically ill patients who were not affected by COVID-19 and a healthy control group, the study protocol was amended twice during the investigation (Ethics Committee number: 20-9242-BO, with amendments on September 2 and September 23, 2021). Figure 1 provides a structured overview of the study process. The study adhered to the principles outlined in the Declaration of Helsinki and received approval from the Ethics Committee of the Medical Faculty of the University of Duisburg-Essen. It was registered in the German Clinical Trials Registry (DRKS-ID: DRKS00022441). Recruitment spanned from April 2020 to July 2023 at the intensive care unit of the Department of Anesthesiology and Intensive Care Medicine, University Hospital Essen, University of Duisburg-Essen.

Skin HSI measurements adhered to standardized conditions, conducted within the first 24 h after ICU admission. Further details on measurements can be found in Kuhlmann et al. [12]. All groups were measured under the same environmental conditions and room lighting was dimmed according to the system’s integrated stray light warning system. In healthy volunteers, a single skin HSI examination of the palm was performed analogous to the examinations in critically ill patients. The SOFA score (SOFA) was modified to consider sedated patients in the ICU [19]. To reflect the assessment of disease severity in ARDS patients undergoing extracorporeal membrane oxygenation (ECMO) treatment, we have added an additional point to the SOFA score in the lung region in the presence of ECMO therapy, increasing the theoretical maximum score from 24 to 25 [12].

### Hospital setting and patients recruitment

In an initial recruitment phase, we were able to include 52 seriously ill septic COVID-19 patients. Most of the critically ill COVID-19 patients required venovenous ECMO therapy with a high SOFA score. However, due to two patients not receiving HSI within the initial 24 h of ICU admission, the sample size for this secondary analysis was adjusted to 50 patients with COVID-19. A detailed description of the COVID-19 patients is provided by Kuhlmann et al. [12]. During a second recruitment phase, we were able to include 25 additional patients with sepsis without COVID-19. In addition, 30 healthy controls were included (see Fig. 1). The number of patients with sepsis and the healthy control group for the secondary analysis was chosen based on feasibility and practicability.

All patients or their legal guardians gave their consent to participate in the study. In cases where patients without legal guardians were unable to give consent, an independent medical advisor agreed to their participation in the study, following local institutional regulations. After their recovery, patients who were previously unable to give consent were asked to participate in the study. Exclusion criteria were patient refusal, expected discharge from the ICU within 72 h of admission, pregnancy, a palliative care approach or imminent death of the patient. In addition, our control cohort consisted of hospital staff from our department, all of whom willingly participated in the study.

### Hyperspectral imaging and feature extraction

HSI was performed using the CE marked TIVITA® Tissue System (Diaspective Vision GmbH, Am Salzhaff, Germany), which is a medical Class I product. The operating principle, technical specifications and data analysis of the HSI camera system are explained in detail by Holmer et al. [13, 15]. The system was used according to producer operating instructions.

Additional to a Red–Green–Blue (RGB) image, the camera system records a spectrum for each pixel, covering wavelengths from 500 to 1000 nm with a spectral resolution of 5 nm, within a spatial resolution of 640 × 480 pixels, resulting in a three-dimensional data cube with 100 measured wavelengths per pixel. From this hyperspectral data cube, a set of four parameters is generated by processing the spectrum within specified wavelength ranges, utilizing only selected spectral information. Each parameter is represented as a two-dimensional image [13, 14].

**Fig. 2:**
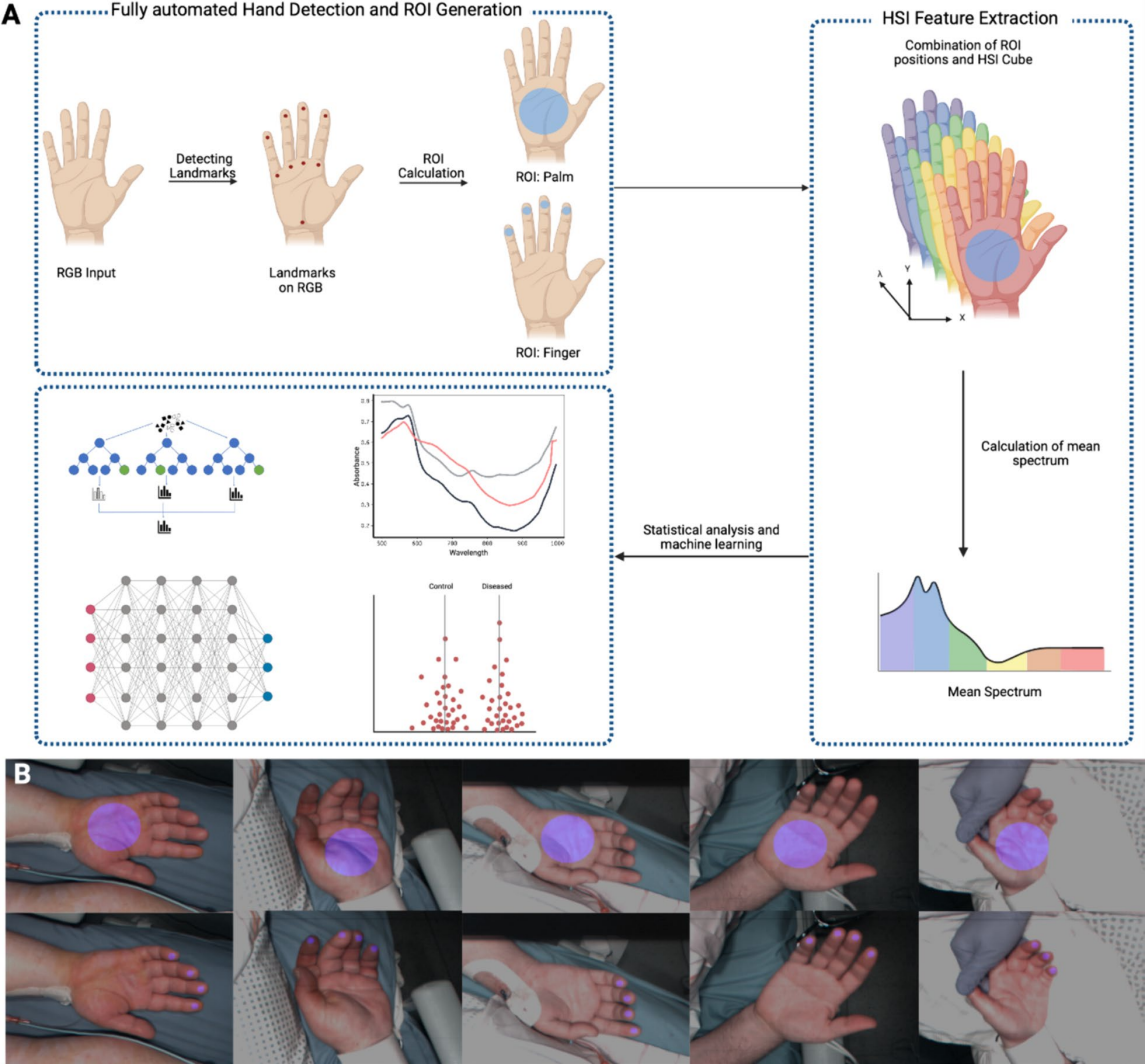
**A** Overview of the methodical procedure. Landmarks are detected on RGB hand images as reference points using the MediaPipe network. These reference points are utilized to generate ROI on the hand. The positions of the ROIs are transferred into the HSI cube, and the average spectrum of pixels is computed. This spectrum can be analyzed and utilized for machine learning inquiries. **B** Examples of hands with highlighted ROIs for palm and fingertips across different patients

For this study we developed an approach to automatically detect and localize regions of interest (ROIs) in RGB images. After this first step, we seamlessly extract features from the raw HSI data by utilizing the ROIs identified in the RGB image. This process involves a seamless transfer of information from the RGB image into the HSI data cube, as shown in Fig. 2.

To identify the anatomical landmarks, MediaPipe's [20] landmark detection was used. We defined circles in the fingertips and palm as representative ROIs. The corresponding anatomical reference points are listed in Table 1.

**Table 1:** The anatomical landmarks for Region of Interest generation by Mediapipe for the palm and fingertips

Region of interest	Landmarks
Fingertips	Fingertips of the pinky, ring, middle, and index finger
Palm	Wrist, metacarpophalangeal joint (MCP) of the index finger and the pinky

**Fig. 3:**
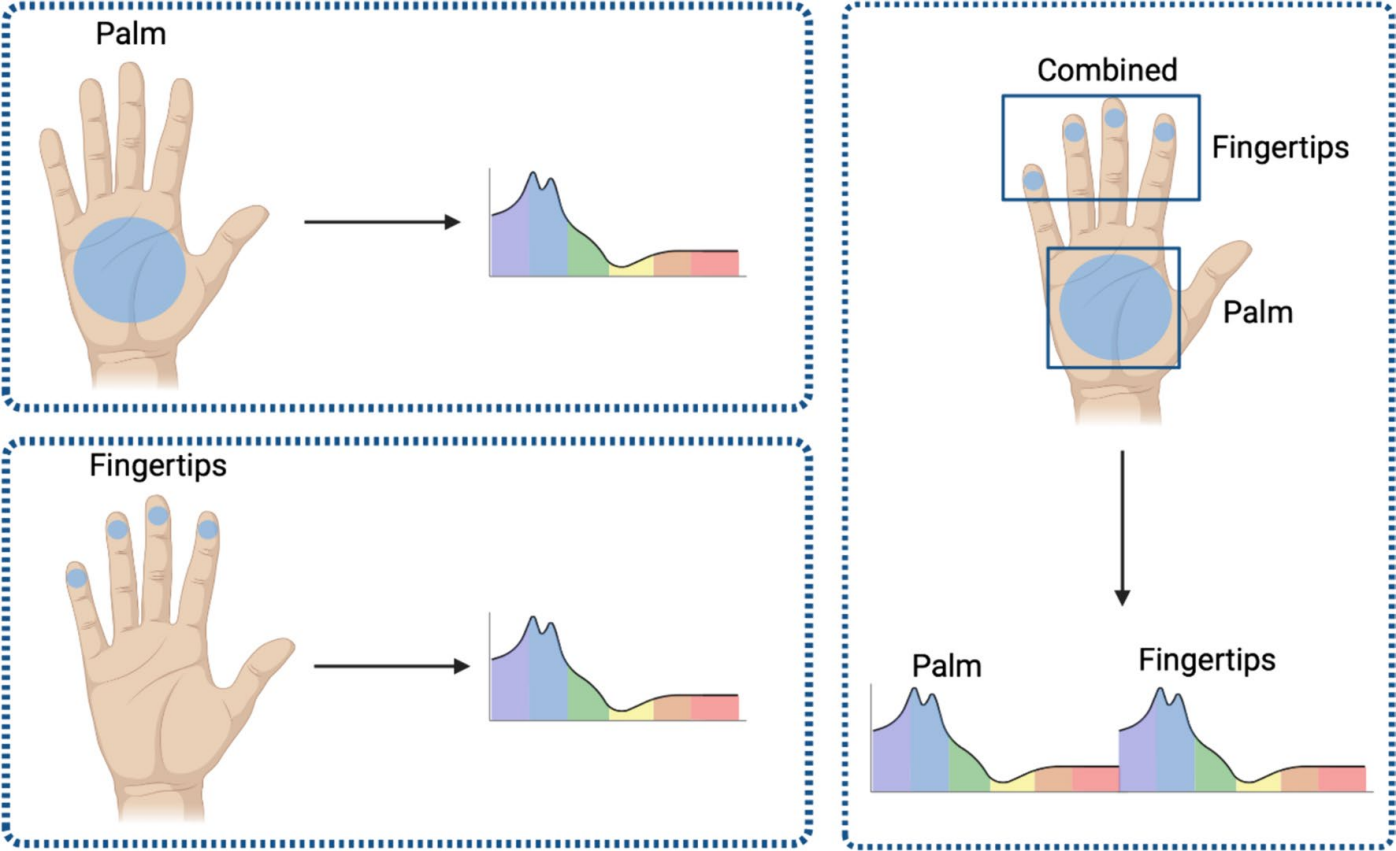
Each ROI covers wavelengths from 500 to 1000 nm with a spectral resolution of 5 nm, resulting in 100 measured wavelengths. The combination of the two ROIs results in a spectrum of 200 wavelengths

The generated landmarks enable the automatic generation of ROIs with any combination of reference points. For the fingertips, the detected landmarks were defined as center points and the radius of the ROI was set to 10 pixels. For the palm, the center point was determined from the triangle of the wrist and the two metacarpophalangeal joint landmarks, and the radius was set to 75 pixels. To exclude pixels in the ROIs that contain interfering factors such as patches or oxygen clamps, a color-based determination was applied and only pixels that capture the skin were considered. Based on the generated ROIs, specific features such as the mean value and confidence intervals across all pixels were calculated for each wavelength, resulting in a corresponding spectrum. Additional to the fingertips and palm ROIs, by combining the fingertips and the palm, we obtained an ROI with a total of 200 wavelengths (shown in Fig. 3).

### Statistical methods

Statistical analyses were performed using Python 3.9.16 and packages SciPy (version 1.10.0) and Scikit-learn [21, 22]. BioRender.com was used to create schematic figures. The mean was used to analyze the distribution of the spectral data of the ROI. Normal assumption was checked using the Shapiro–Wilk test. For data with normal assumption, the mean and standard deviation were reported. Otherwise, the median value with interquartile range (IQR) was used. In addition, to check whether the absorption spectra between the patient groups differ significantly, the Mann–Whitney-U Test was performed. For a *p*-value below 0.05, it was assumed that the spectra differed significantly at these wavelengths. To control for false discovery rate due to multiple comparisons, the p-values obtained from the Mann–Whitney-U Test were further adjusted using the Benjamini, Krieger, and Yekutieli (BKY) approach, as implemented in the statsmodels package [23]. The results are visually depicted in bar plots, indicating significant (yes) and non-significant (no) differences. Difference plots were created by calculating the difference in absorption between the mean spectra of the patient groups for each wavelength. These differences were then represented as bar plots.

### Machine learning

For the classification of the disease state a Random Forest (RF) Classifier was used. The model default hyperparameters were used, except for the maximum depth of the trees which was set to 30. The model training was performed using Leave-One-Out cross-validation (CV), resulting in a total of 104 classifiers, which were then combined into an ensemble model. This ensemble model combines the predictions from the different CV runs using the mean, to obtain a robust estimate of model performance. The metrics used for evaluation were area under curve (AUC), F1-score, and 95%-confidence interval. The F1-score is based on the associated precision and sensitivity of the prediction model, where the score can range between 0 and 1. It is particularly suitable in the case of imbalanced data sets [24]. The model performance was visualized by a receiver operating characteristic (ROC) curve and feature importance was determined by Mean Decrease in Impurity (MDI) to identify relevant wavelengths for the model decision.

### Patient groups

Firstly, we investigated whether the spectral data pattern could be used to differentiate between healthy controls and septic patients. For this purpose, the data of all septic patients (n = 75) were summarized and compared with healthy controls (n = 30). To investigate whether the raw spectra allow conclusions regarding the disease severity, we arbitrarily defined two groups post hoc based on their SOFA Score: less severely ill (SOFA ≤ 5) and severely ill (SOFA > 5), each compared to the healthy control group (see Table 2).

**Table 2:** Patient characteristics

	Patients with sepsis			Healthy controls
	All	SOFA ≤ 5	SOFA > 5	
Number of patients	n = 75	n = 13	n = 62	n = 30
Sex [female]	25/75 (33%)	9/13 (69%)	16/62 (26%)	16/30 (53%)
Age, years	59.4 (± 13.1)*	60.5 (± 12.2)*	58.2 (± 13.2)*	48.6 (± 11.8)*
SOFA-Score	11.0 (± 4.5)*	3.3 (± 1.1)*	12.6 (± 3.0)*	–
ECMO [yes]	45/75 (60%)	1/13 (8%)	44/62 (71%)	–

Sex, age, SOFA score and ECMO status of all included patients (n = 105). Values are expressed as * mean (± standard deviation)

## Results

### Cohort characteristics

We analyzed the skin HSI images from 75 patients with sepsis and from 30 healthy controls. The characteristics of the 105 patients (age: 60 ± 17; female: 39%) are summarized in Table 2.

### Disparities in HSI spectral raw data between healthy individuals and patients with sepsis

**Fig. 4:**
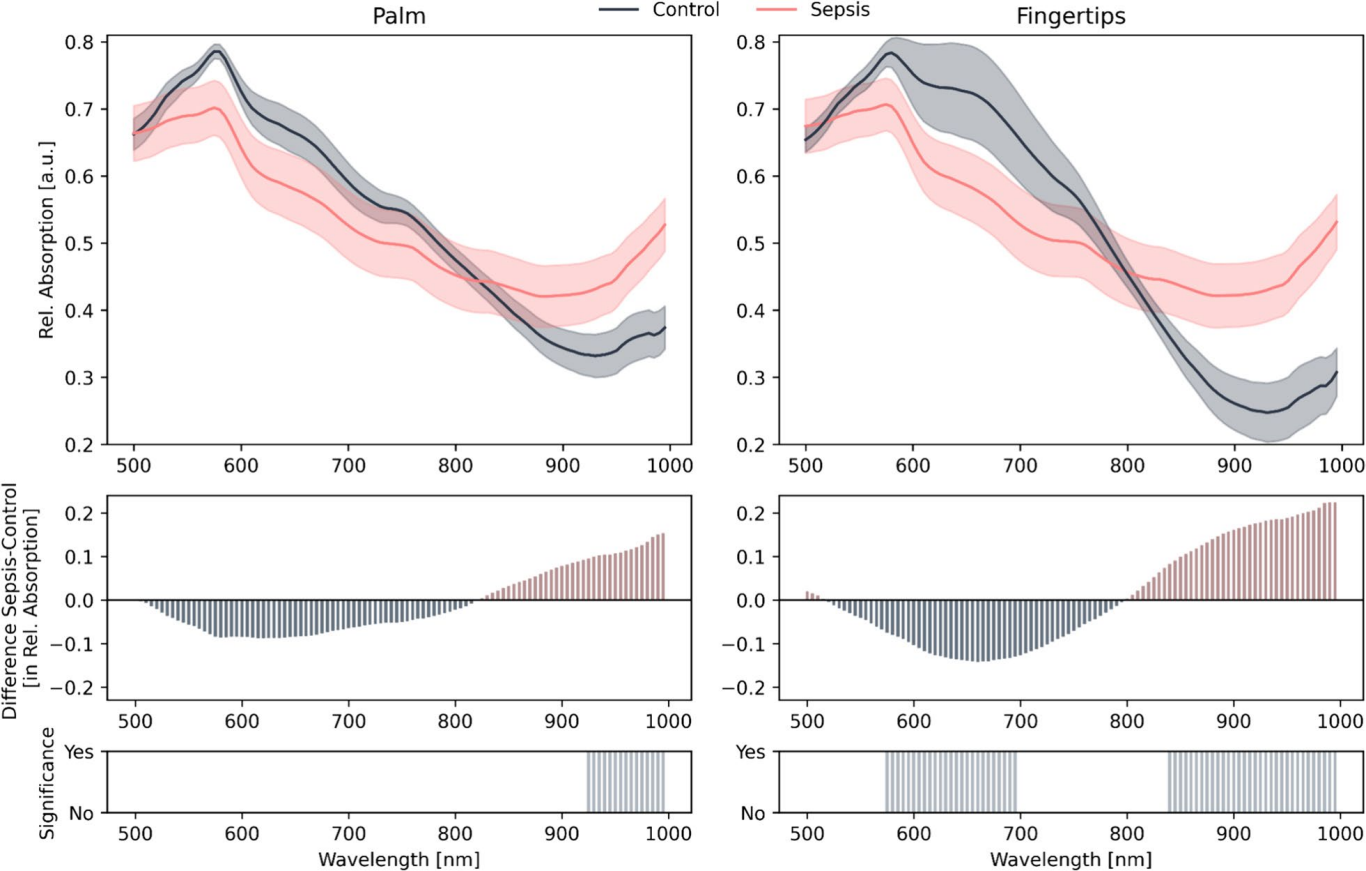
Mean Spectra with 95% Confidence intervals for control and for septic patients. Comparison of the spectra from healthy control (dark blue) and patients with sepsis (pink) for two distinct ROI: palm (left) and fingertips (right). In the middle, the difference between sepsis and control for each wavelength is shown. The colors in the difference plots are chosen such that the color indicates which spectrum (with the same color) exhibits higher absorption. Bar plots positioned below highlight significant differences between the spectral distributions of the groups, showcasing the discriminative spectral bands. Differences were determined to be statistically significant using the Mann–Whitney-U Test, with a *p*-value below 0.05. Test correction was performed using the BKY approach

The spectral pattern of healthy controls differs from that of patients with sepsis, as can be seen in the difference plot of Fig. 4.

For Fingertip ROI, it was shown that in the range of 575–695 nm, a significantly lower absorption was found in patients with sepsis compared to the healthy control. For the palm, there are no significant differences between these spectra in this wavelength range. In contrast, the spectra of wavelengths from 925 to 1000 nm for the Palm ROI and from 840 to 1000 nm for the Fingertip ROI differ significantly. Comparing the two ROIs, the spectra of the healthy controls and patients with sepsis differ at the fingertips for more wavelengths overall. In addition, the spectrum of the healthy controls shows significantly lower absorptions at wavelengths between 900 and 1000 nm for the fingertips as ROI compared to the palm. The spectral characteristics of the healthy controls and the patients with sepsis are shown in Fig. 4.

### Severely ill sepsis patients show greater HSI spectral differences compared to healthy individuals than less severely ill patients

**Fig. 5:**
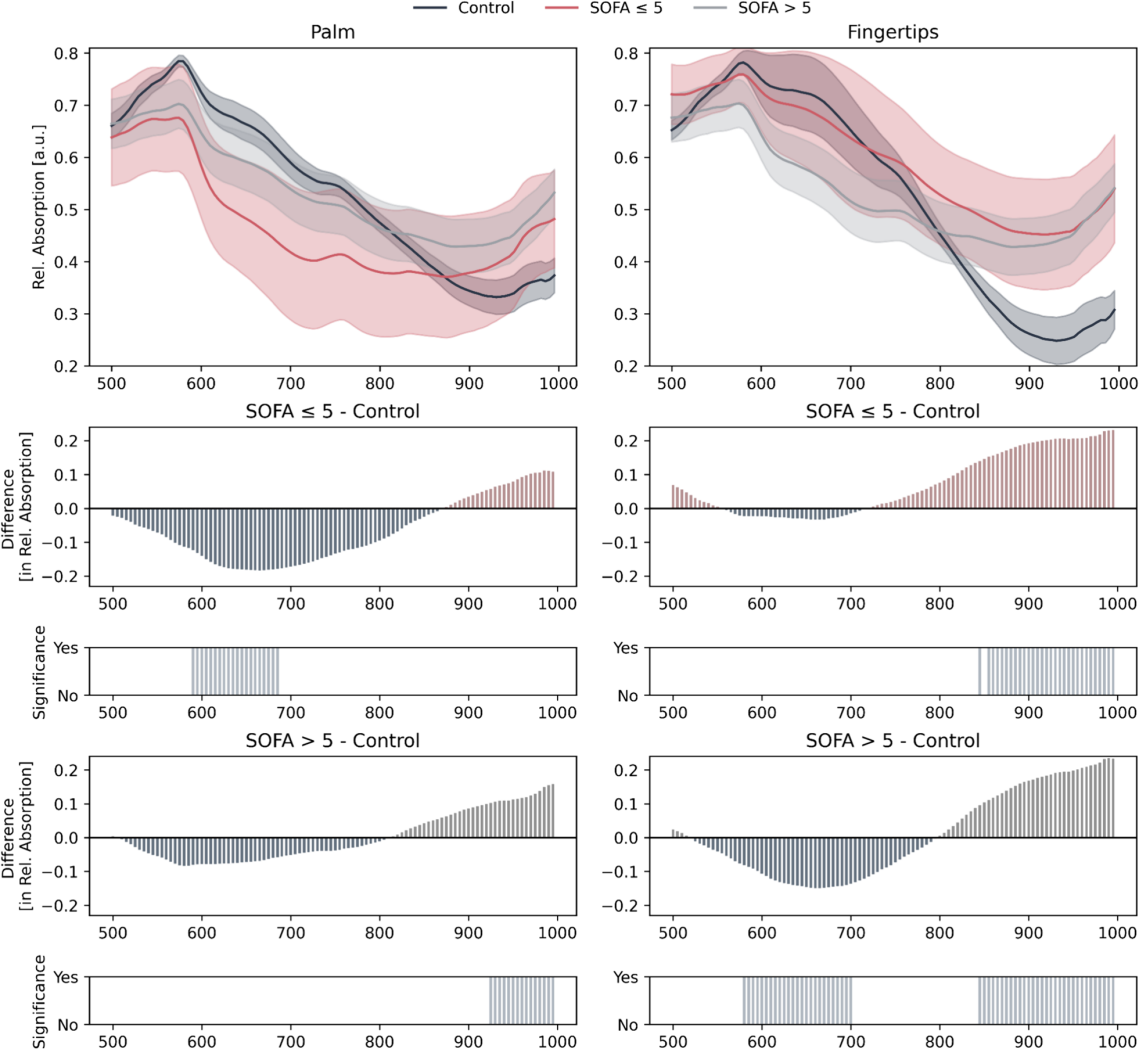
Mean Spectra Comparison with 95% Confidence Intervals. Comparison of the spectra for the palm and fingertips across three distinct patient groups: SOFA-score: healthy control (black), less severe organ dysfunction = SOFA ≤ 5 (dark blue) and severe organ dysfunction = SOFA > 5 (red). Below the spectra, difference plots between the patients with sepsis and the healthy controls are shown as bar plots, as well as bar plots highlight significant differences between the spectral distributions of the groups, showcasing the discriminative spectral bands. Differences were determined to be statistically significant using the Mann–Whitney-U Test, with a *p*-value below 0.05. Test correction was performed using the BKY approach. The colors in the difference plots are chosen such that the color indicates which spectrum (with the same color) exhibits higher absorption

The spectra of the HSI captured from the palm and the fingertips from both healthy control subjects and patients diagnosed with sepsis, categorized by disease severity is presented in Fig. 5.

The confidence intervals of the spectra of patients with less severe organ dysfunction differed from those of healthy controls in the palm area at 590–685 nm and for the fingertips at 845–1000 nm. In contrast, the confidence intervals for the sepsis patients with more severe organ dysfunction differed from the healthy controls in the palm of the hand at 925–1000 nm and at the fingertips of 580–700 nm, and of 845–1000 nm. There are no significant differences between the spectra of less severely organ dysfunction and severely organ dysfunction patients.

### HSI enables reliable differentiation between critically ill patients with sepsis and healthy individuals

**Fig. 6:**
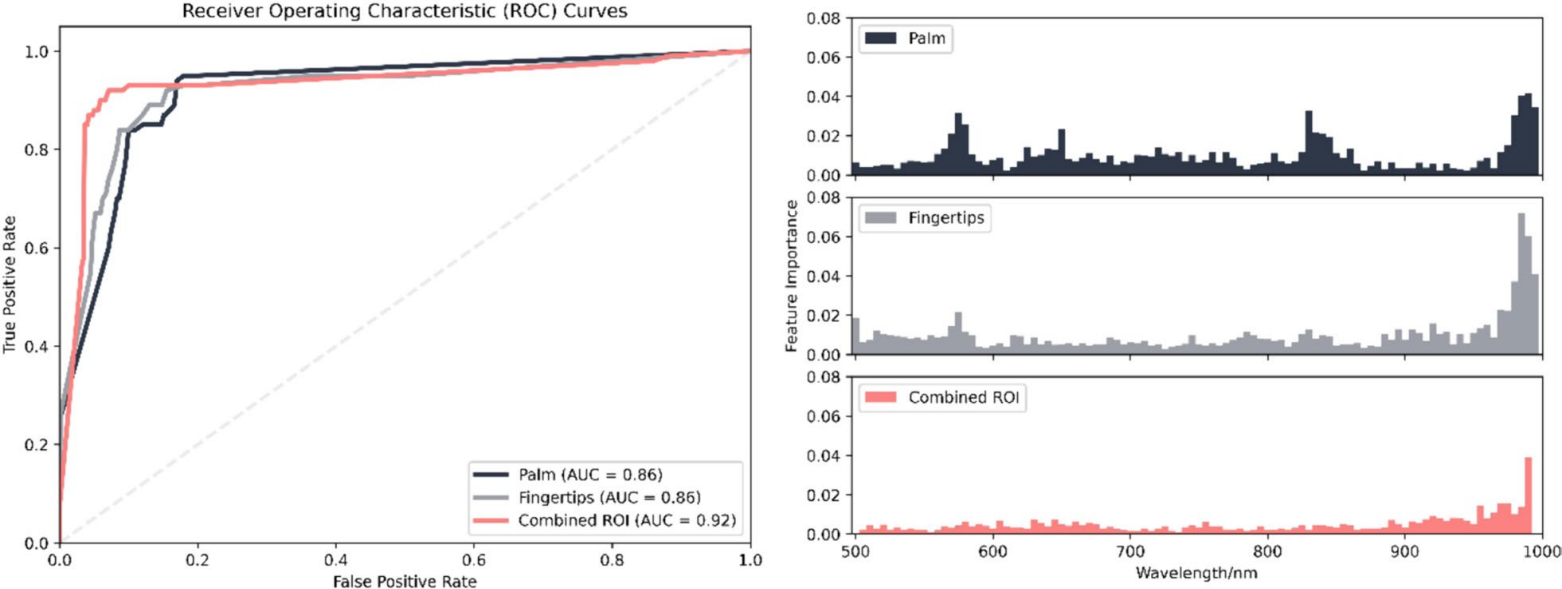
Classification results of healthy control and septic patients for palm (dark blue), fingertips (grey) and combination of both (pink) ROI. The ROC curves of the three ROIs are shown on the left, and on the right are the feature importance plots showing which wavelengths were relevant for the classification. For the combined ROI, the mean feature importance of palm wavelengths and fingertips wavelengths was calculated

Assessing the model's capability to discriminate between the healthy control and septic patients, ROC curves were generated for all three ROIs (Fig. 6).

Comparable AUC values were found for the palm (AUC = 0.86, CI = 0.82;0.93) and fingertips ROIs (AUC = 0.86, CI = 0.82;0.94). The palm demonstrated a better F1 score than the fingertips (F1 = 0.94 vs.0.92). The combination of palm and finger showed the most promising results (AUC = 0.92, CI = 0.87;0.97, F1 = 0.92). Regarding the feature importance plots, in the case of the combined ROI, the mean of the relevancies for the palm wavelengths and fingertips wavelengths was calculated. The analysis of the feature importance diagrams shows that the most important wavelengths to distinguish between septic patients and healthy controls compared to all wavelengths are the same for all ROIs, covering the wavelengths from 960 to 1000 nm. In addition, the wavelengths from 560 to 590 nm were also important for the prediction for both the palm and the fingertips. This is consistent with the significant differences in the spectral properties shown in Fig. 4.

The classification of disease severity showed the highest performance in the combined ROI, particularly for control vs. severe organ dysfunction (AUC = 0.92, CI = 0.87;0.94, F1 = 0.91). The palm region also performed well for this classification (AUC = 0.90, CI = 0.87;0.95, F1 = 0.88). Fingertips had moderate performance for control vs. less severe (AUC = 0.72, CI = 0.47;0.83, F1 = 0.60) and severe organ dysfunction (AUC = 0.80, CI = 0.76;0.91, F1 = 0.71). For all models the performance improved if both ROI were combined. However, distinguishing less severe vs. severe organ dysfunction was challenging across all regions, with lower scores, particularly for the palm (AUC = 0.45; CI = 0.40;0.63, F1 = 0.80) and fingertips (AUC = 0.45, CI = 0.35;0.58, F1 = 0.74).

## Discussion

To the best of our knowledge, we present here the first results of an automated extraction of skin HSI raw data from critically ill patients with sepsis. The machine learning-based analysis of these data allowed a clear distinction between patients suffering from sepsis and a healthy control group. The analysis of the feature importance diagrams shows that in all ROIs the differences in the wavelengths from 550 to 600 nm and from 960 nm most reliably discriminate between patients with sepsis and healthy controls.

Despite extensive research on monitoring technologies, bedside microcirculatory monitoring has yet to be routinely integrated into clinical practice in critical care medicine. Our findings for automated skin HSI analysis may be part of the solution for the development of comprehensive, automated, operator-independent, and objectively assessable bedside microcirculatory monitoring as advocated by Duranteau et al. [3].

Skin mottling and capillary refill time are widely recognized clinical indicators of shock, highlighting the skin’s relevance as an important organ for research in microcirculatory monitoring [5, 25–28]. Skin HSI is a novel non-invasive optical imaging technology for microcirculatory monitoring in critical care. HSI generates characteristic optical tissue patterns, known as spectral signatures or fingerprints, that allow qualitative and quantitative differentiation between tissue types and changes in tissue composition, including pathological changes [8, 13–15]. HSI shares some technological capabilities and limitations of near infrared spectroscopy (NIRS), which has been extensively studied as a microcirculatory monitoring tool. Besides the use of different spectral ranges between NIRS and HSI, a major difference is that NIRS is continuous whereas HSI represents an intermittent optical monitoring method [29, 30]. The use of ML to analyze HSI data is growing, improving diagnostic accuracy and disease classification in a variety of medical fields, including gastric, brain, and skin cancer detection, as well as eye disease or image-guided surgery [7, 31–33]. In contrast to other microcirculatory monitoring methods such as sublingual video microscopy (SVM) [3], HSI does not allow visualization and direct flow measurement in the microcirculation. HSI, on the other hand, provides detailed spatial information about the oxygen supply and tissue perfusion as well as the tissue water content in the targeted area [8, 13–15]. In addition, an HSI measurement can be carried out quickly and provides a reliable and objective assessment of the microcirculation within a few seconds, particularly when caring for critically ill patients in intensive care units and emergency departments. Previous studies by Dietrich et al., Kazune et al. and Kuhlmann et al. demonstrate that HSI can differentiate between healthy controls and septic patients, revealing a characteristic heterogeneous skin oxygenation pattern in septic patients. Additionally, HSI has been proposed to provide microcirculatory feedback to access macrocirculatory measures and detect adverse side effects of vasopressor and fluid therapy [9, 11, 12, 16, 17].

One hypothesis for this proof of concept study was that by developing an automated image analysis pipeline with ML analysis using raw spectral data from the TIVITA camera system, more detailed information about relevant spectral wavelengths ranges could be obtained that would allow us to more specifically describe a "septic" spectral signature of skin HSI at the palm or/and fingertips.

There is currently no consensus on the appropriate anatomical site for skin HSI studies to assess microcirculatory function. The palm as well as the fingertips have been previously proposed by Dietrich et al. and Kuhlmann et al. to possess primarily clinical advantages as evaluation sites in critical ill patients [9, 12]. Contrary, Kazune et al. performed HSI examinations above the kneecap to determine correlations of HSI with mottling score [11, 16]. The observed differences in the spectral profiles between the palm and the fingertips in our study indicate that each measuring site has specific characteristics that must be considered for data interpretation. A key finding of our machine learning analysis is that the combined evaluation of the palm and fingertips is the most meaningful in terms of the appropriate measurement location. This also supports the relevance of an automated image analysis that allows standardized and combined data analysis of two measurement sites.

We observed an overlap of spectral profiles between septic patients and healthy controls for both measuring sites. Similar overlaps between pathological values and healthy control values are reported for NIRS parameters in sepsis too [5, 34]. This indicates that the normal value ranges for skin HSI need to be further specified in follow-up studies. In addition to the evaluation of "static" parameters, this will include the dynamic assessment of changes in the spectral profile during microvascular provocation tests, such as vascular occlusion tests, or during hemodynamic treatment of critically ill patients using bedside skin HSI.

Interestingly our analysis demonstrated the spectral profiles and relevant wavelength ranges changed with increasing disease severity and depending on the measurement site. Patients with sepsis displayed a spectral profile that is indicative of impaired tissue oxygenation combined with an increase in tissue water content. Specifically, we found the highest number of significantly different wavelengths (580–700 nm and 845–1000 nm) in more severely ill patients (SOFA score > 5) when measured at the fingertips. In the future, a fully automated skin HSI analysis could serve as a bedside tool in fluid therapy management by integrating the quantification of changes in tissue water content tissue edema combined with perfusion/oxygenation variables, thus preventing detrimental effects of fluid therapy.

It should be noted that the results shown in this study are just the beginning of research into machine learning-based HSI analysis at the point of care using the raw spectrum. We hypothesize that with a significant increase in the number of cases and a simultaneous increase in the number of diseases, further effects between wavelengths may occur, which could not yet be investigated in the context of this study. This can be seen in the approach through the feature importance of the machine learning models. Even if not all wavelengths were significantly different in the statistical analysis, these non-significant wavelengths still have an albeit smaller significance for the decision of the model. This potentially provides greater scope for differentiating between disease groups when focusing on using the full HSI spectrum [35]. Another future methodological opportunity lies in the use of deep learning techniques. In our study applying ML to HSI data from septic and healthy controls, we found that traditional models such as Random Forest are just the beginning. While those models have shown promise in dealing with the complexity of HSI data through a preceding feature extraction, the high dimensionality inherent in HSI images presents an opportunity for more advanced techniques. Deep learning methods, particularly convolutional neural networks (CNNs), could significantly improve classification tasks due to their ability to automatically extract and learn complex features from high-dimensional data [7]. These techniques have already been used in combination with HSI data for other medical research questions, such as Seidlitz et al. [35] and Maktabi et al. [36], demonstrating their potential in various applications [7]. However, it is important to note that deep learning approaches require larger sample sizes to achieve optimal performance and generalisability. Therefore, the exploration of deep learning approaches is crucial to advance the analysis and interpretation of HSI data in clinical applications.

In addition, HSI data can be seamlessly accessed and exchanged using the raw data format or data standards like DICOM. This facilitates direct transfer and enables immediate access for data analysis through ML pipelines like the presented proof of concept. This potentially would make HSI analysis results available to clinicians immediately after creation and in near real-time. Also, recent developments of more leight-weighted and smaller HSI devices [37, 38], further drive wider applicability and clinical application. At the same time, the setup described with the potential technical enhancements also would enable a more efficient and routine friendly HSI monitoring of patients.

A comprehensive evaluation of microcirculatory alterations in critically ill should conceptually include the evaluation of microcirculatory flow and functional capillary density together with tissue oxygenation monitoring. Hilty et al. used a machine learning-based analysis of the microcirculation of the tongue to distinguish between severely ill COVID-19 patients and healthy volunteers in a study with 157 patients [6]. ML analysis of SVM image data enabled reliable differentiation between healthy controls and COVID-19 patients (AUC 0.75) [6]. By combining data from the palm and fingertip, we achieved reliable differentiation between sepsis patients and healthy controls, with an AUC of 0.92. The wavelengths 970–1000 nm (palm) and 960–1000 nm (fingertip) were essential for feature importance and machine learning-based discrimination. An important next step would therefore be a combined ML-based investigation of flow-based microcirculation monitoring such as SVM together with optical monitoring with skin HSI and assessment of tissue oxygenation quality and changes in tissue water content.

The values of different devices for HSI have not yet been compared and standardized spectral ranges as well as uniform image acquisition technologies and analysis software algorithms are lacking. In particular, the robustness and generalisability of HSI data is still an unresolved problem [8, 9, 12, 18]. This complicates comparability and leads to inconsistent results in different HSI studies [8, 9, 16–18]. The automatic generation of ROI and HSI analysis presented here enhances the consistency and technical reliability of the overall assessment process. Automated image analysis and artificial intelligence could reduce observer bias and help guide microcirculation-targeted diagnostic and therapeutic decisions. This is further supported by the proposed pipeline, which enhances robustness by excluding potential sources of noise, such as adhesive bandages or oxygen patches within the ROI. This method could facilitate the comparison of HSI data between different disease entities and patient cohorts.

Several limitations should be noted when interpreting our results. Although no patients with dark skin color participated in our study, it is important to acknowledge the possible influence of this factor on HSI measurements as a limitation. In addition, it should be noted that individual skin conditions, such as differences in epidermal thickness, can influence the results obtained by skin HSI analysis. For this proof of concept approach, we did not analyze in depth other factors influencing microcirculation such as ECMO, catecholamines or fluid therapy, nor did we analyze which SOFA subcategory has the strongest influence on skin HSI measurements. Additionally, patient-specific factors like age, gender and preexisting health conditions which could affect microcirculation, were also not investigated. All patients included in this study were suffering from sepsis. For this proof of concept approach, we did not investigate whether there were differences in the skin HSI profile between patients with and without Covid-19-associated sepsis. Another limitation of the study is that the HSI dataset used in this study is comparably small. To integrate the presented proof of concept methods into clinical applications, larger and more diverse datasets are needed to place the results in a broader context. This is also applicable for the disease severity groups in the current dataset which should be investigated in future studies with more balanced and larger datasets. As more data becomes available, the potential for ML and deep learning techniques will improve, allowing a more thorough analysis of spectral information in terms of disease and disease severity.

## Conclusion

A key future challenge in the care of critically ill patients is to develop a non-invasive, simple and accurate method for assessing microcirculation. This proof of concept study demonstrates for the first time that integrating automated and standardized ROIs with automated skin HSI enables effective discrimination between healthy individuals and patients with sepsis. The combination of automated ROIs and advanced machine learning algorithms applied to raw HSI raw data might offer an efficient initial assessment of microcirculation, facilitating the rapid identification of critically ill patients for timely intervention and improved clinical management.

## Data Availability

The datasets generated during and/or analysed during the current study are available from the corresponding author on reasonable request.
